# Phase II study of avelumab and trastuzumab with FOLFOX chemotherapy in previously untreated HER2-amplified metastatic gastroesophageal adenocarcinoma

**DOI:** 10.1093/oncolo/oyaf195

**Published:** 2025-07-17

**Authors:** Michael S Lee, Joseph Chao, Mary F Mulcahy, Pashtoon M Kasi, Angela T Alistar, Sarbajit Mukherjee, Mehmet Akce, Dominic T Moore, Autumn J McRee, Ashwin Somasundaram

**Affiliations:** Department of Innovative Medicine Research & Development, Johnson and Johnson Innovative Medicine Research & Development, LLC, Spring House, PA, USA; Department of Medicine, City of Hope Comprehensive Cancer Center, Duarte, CA, USA; Department of Medicine, Northwestern University, Chicago, IL, USA; Department of Medicine, City of Hope Comprehensive Cancer Center, Duarte, CA, USA; Department of Medicine, Morristown Medical Center, Morristown, NJ, USA; Department of Medicine, Roswell Park Cancer Center, Buffalo, NY, USA; Department of Medicine, University of Alabama at Birmingham, Birmingham, AL, USA; University of North Carolina Lineberger Comprehensive Cancer Center, Chapel Hill, NC, USA; Department of Innovative Medicine Research & Development, Johnson and Johnson Innovative Medicine Research & Development, LLC, Spring House, PA, USA; University of North Carolina Chapel Hill, Department of Medicine at Chapel Hill, NC, USA

**Keywords:** HER2, trastuzumab, gastric cancer, esophageal cancer, immunotherapy

## Abstract

**Background:**

Trastuzumab and multiagent chemotherapy have been the standard of care for the 20-30% of metastatic gastric and esophageal adenocarcinomas that overexpress HER2. Preclinical data show that trastuzumab requires a functional adaptive immune system for efficacy, suggesting synergy of trastuzumab combined with immune checkpoint inhibitors, further supported by current clinical studies.

**Methods:**

HCRN GI17-319 was a multicenter, single-arm, phase II clinical trial with a prespecified 6-subject safety run-in of the anti-PD-L1 antibody avelumab, combined with trastuzumab and mFOLFOX6, in previously untreated, metastatic, HER2-amplified gastric and esophageal adenocarcinomas. The primary endpoint was the best overall response within 24 weeks. Subjects received 9 cycles of induction avelumab, trastuzumab, and mFOLFOX6, followed by maintenance avelumab + trastuzumab. The study was initially designed as a Simon’s 2-stage trial, but enrollment was stopped after the 18-subject first stage for reasons unrelated to safety or efficacy.

**Results:**

A total of 18 subjects, including the 6-subject safety run-in, were enrolled 4/2019-8/2020. The 24-week response rate was 11/18 (61%; 95% CI: 39%-84%), and the confirmed overall response rate is 9/18 (50%). With a median follow-up of 14.6 months, the median PFS was 8.0 months (95% CI: 5.3-NA) and median OS was 13.1 months (95% CI: 11.5-NA). The regimen was well tolerated, without any new safety signals.

**Conclusions:**

The combination of avelumab, trastuzumab, and FOLFOX chemotherapy demonstrated some activity, with a reasonable response rate and median PFS. These outcomes provide some support to other clinical trials of similar agents and support the future evaluation of adding avelumab in this setting. NCT03783936.

**Lessons Learned**

- The addition of the anti-PD-L1 antibody avelumab to chemotherapy with FOLFOX + trastuzumab in HER2-overexpressing gastric and esophageal adenocarcinoma yielded a response rate within 24 weeks of 61%, which is reasonable given the findings of the ToGA trial, which yielded a response rate for chemotherapy + trastuzumab of 47%.
- Our study showed a median PFS of 8.0 months and a median OS of 13.1 months. This corroborates the KEYNOTE-811 phase III trial, in which the addition of pembrolizumab to trastuzumab and chemotherapy doublet yielded a confirmed response rate of 74.4%.

## Trial information

**Table:** 

Trial information	
**Disease**	Eligible patients had histologically confirmed esophageal, gastroesophageal junction, or gastric adenocarcinoma that was unresectable or metastatic, with HER2 amplification assessed by local pathology laboratory assessment (3 + by immunohistochemistry, or 2 + by immunohistochemistry with *in situ* hybridization with HER2/CEP17 ratio ≥2),
**Stage of disease/treatment**	IV
**Prior therapy**	None
**Type of study**	Multicenter, single-arm, investigator-initiated phase II clinical trial conducted at 7 academic and community centers in the United States.
**Primary endpoints**	The primary endpoint was the best objective response rate per RECIST v1.1 by 24 weeks.
**Secondary endpoints**	Secondary endpoints included PFS per RECIST 1.1, overall survival (OS), and safety and tolerability, assessed by adverse events in CTCAE v5.0.
**Additional details of endpoints or study design** The study was designed with α = 0.05 and 80% power to detect improvement in response rate from a null hypothesis of 47% to an alternative hypothesis of 65%. This would have required a total of 57 subjects; 18 subjects would be enrolled for the first stage, and if there were ≥10 responses, then the study would proceed to completion of enrollment and enroll another 39 subjects. However, after the first stage of the study was completed, the study was halted due to slower-than-expected enrollment, and this was not due to efficacy or toxicity concerns. The Kaplan–Meier method was used to estimate the time to event functions of OS and PFS by RECIST 1.1 and iRECIST criteria (PFS and iPFS). OS has been calculated using the start of treatment date to the date of death from any cause, or date of last contact (censored). PFS and iPFS have been calculated using the start of treatment date to either the date of progression (by RECIST 1.1 and iRECIST criteria, respectively) or the date of death from any cause, or date of last contact (censored). The ‘loglog’ method (based on the log of the hazard) was used for calculating 95% confidence intervals for median OS, PFS, and iPFS. All reported *P*-values are 2-sided, with *P*-values less than 0.05 considered significant. Statistical analyses were performed using both SAS (version 9.4, Cary, NC) and R (R Foundation for Statistical Computing, Vienna, Austria; http://www.R-project.org/).	

## Drug information

**Table:** 

Drug information	
**Generic/working name**	Avelumab
**Company name**	EMD Serono
**Drug type**	Immunotherapy (anti-PDL1)
**Drug class**	Monoclonal Antibody
**Dose**	10 mg/kg
**Route**	IV
**Schedule of administration** given every 14 days for all cycles.	

Drug information	
**Generic/working name**	Trastuzumab
**Company name**	Genentech, Inc.
**Drug type**	Targeted therapy (anti-HER2)
**Drug class**	Monoclonal antibody
**Dose**	6mg/kg loading dose, then 4 mg/kg
**Route**	IV
**Schedule of administration** given every 14 days for all cycles (loading dose only in cycle 1).	

Drug information	
**Generic/working name**	5-fluorouracil
**Company name**	Hoffman-La Roche
**Drug type**	Chemotherapy
**Drug class**	antimetabolite
**Dose**	400 mg/m^2^ bolus and 2400 mg/m^2^
**Route**	IV
**Schedule of administration** given every 14 days for cycles 1-9.	

Drug information	
**Generic/working name**	Oxaliplatin
**Company name**	Pfizer
**Drug type**	Chemotherapy
**Drug class**	Alkylating agent
**Dose**	85 mg/m^2^
**Route**	IV
**Schedule of administration** given every 14 days for cycles 1-9.	
**Drug information**	
**Generic/working name**	Leucovorin
**Company name**	Fresenius Kabi, LLC
**Drug type**	Folic acid analog
**Drug class**	Folic acid analog
**Dose**	400 mg/m^2^
**Route**	IV
**Schedule of administration** given every 14 days for cycles 1-9.	

## Patient characteristics

**Table:** 

Patient characteristics	
Number of patients, male	13
Number of patients, female	5
Stage	IV
Number of prior systemic therapies: median (range)	0
Performance status: ECOG 0 or 1	18
Performance status: ECOG 2 or above	0
Cancer types or histologic subtypes	HER2-overexpressing gastroesophageal adenocarcinoma

## Primary assessment method

**Table:** 

Primary assessment method	
**Title**	Response rate
**Number of patients screened**	19
**Number of patients enrolled**	18
**Number of patients evaluable for toxicity**	18
**Number of patients evaluated for efficacy**	18
**Evaluation method**	RECIST 1.1

**Outcome notes**

**Table:** 

Response assessment	N	%	
CR	1	6%	
PR	10	56%	
SD	5	28%	
PD	2	11%	

(Median) duration assessments	#	Day/week/month?	CI
PFS	8.0	months	5.3-NA
OS	13.1	months	11.5-NA

## Discussion

**Figure 1.**
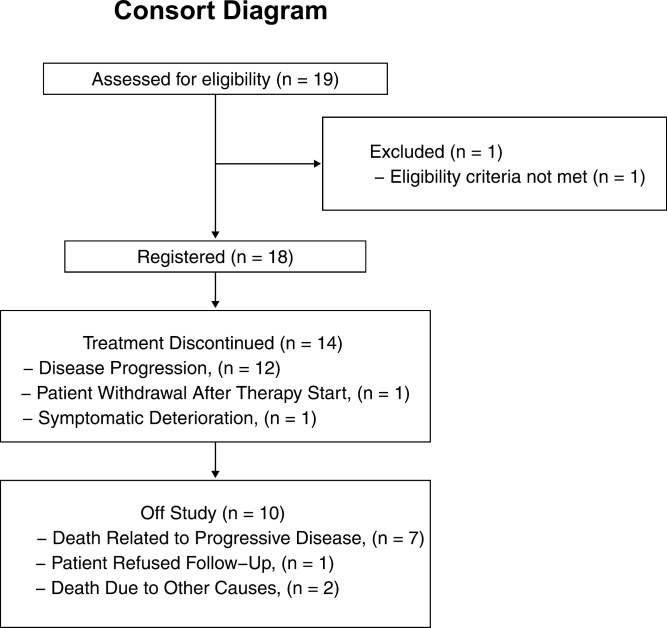
Diagram of patient accrual and summary of patient outcomes, including reasons for treatment discontinuation and removal from study.

**Figure 2.**
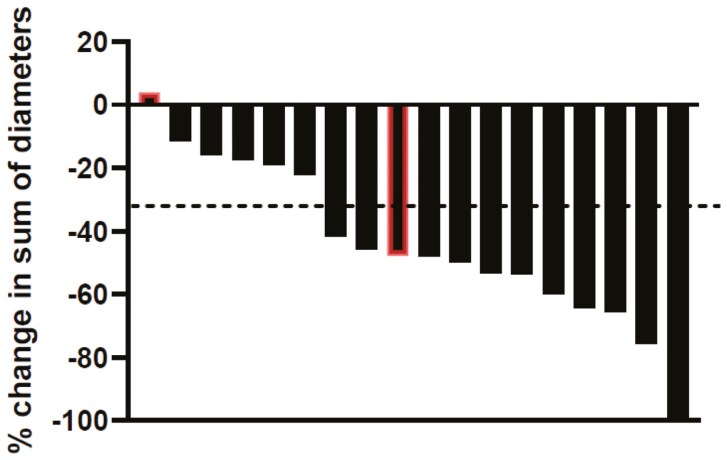
Best change in the sum of diameters of target lesions. Bars outlined in red showed progression in nontarget lesions. CR, complete response; PR, partial response; SD, stable disease. PD: progressive disease.

**Figure 3.**
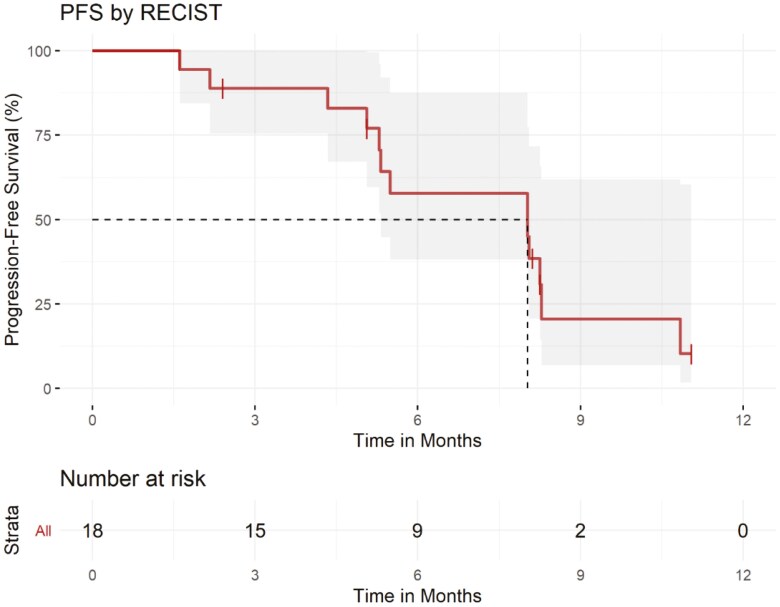
Kaplan–Meier curve of progression-free survival.

**Figure 4.**
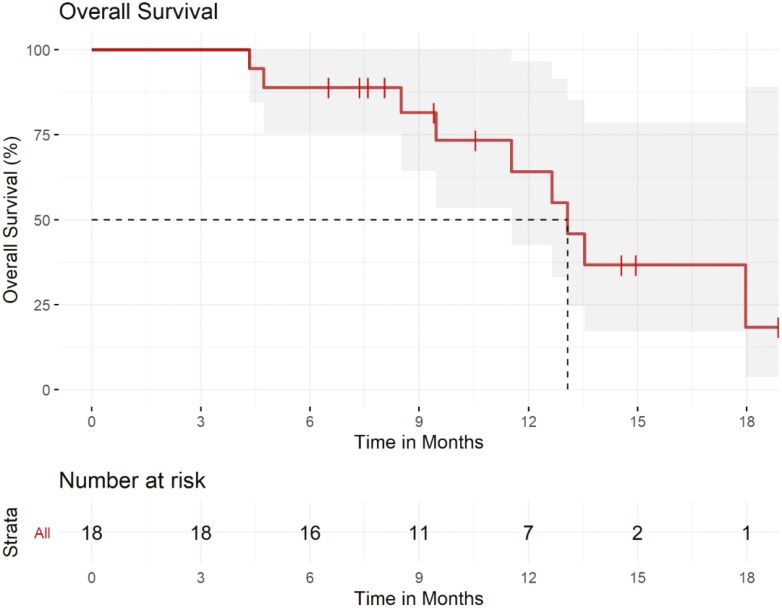
Kaplan–Meier curve of overall survival.

This multicenter, single-arm controlled clinical trial of FOLFOX + trastuzumab + avelumab showed the best objective response rate (ORR) by 24 weeks of 61% among 18 patients, with a favorable safety profile and no unexpected toxicities (Figures 1 and 2 and Tables 1, 2, and 3). While cross-trial comparisons are imperfect with multiple biases and confounding factors, the ORR in our study does have a signal for activity. The median PFS and OS with the regimen in this study were a promising 8.0 months (95% CI: 5.3-NA) and 13.1 months (95% CI: 11.5-NA), respectively (Figures 3 and 4). Thus, these data support larger evaluation of avelumab to standard chemotherapy doublet and trastuzumab regimens in patients with HER2-overexpressing gastroesophageal cancers. Indeed, these results corroborate previously published single-arm phase II clinical trials of chemotherapy + trastuzumab + pembrolizumab, each enrolling 37-43 patients, with ORRs of 77%-91%.^1,2^ These also corroborate the recently presented results from the KEYNOTE-811 phase III clinical trial, in which the addition of pembrolizumab to trastuzumab and chemotherapy doublet (either 5-fluorouracil + cisplatin or capecitabine + oxaliplatin) improved PFS, OS, and confirmed ORR. Notably, the confirmed ORR with the addition of pembrolizumab was an impressive 72.6% (95% CI: 67.6-77.2).^3,4^ More recently, updated results from KEYNOTE-811 were presented, and with a median follow-up of 38.4 months, the median PFS with chemotherapy plus trastuzumab and pembrolizumab was 10.0 months (95% CI: 8.6-12.2), and median OS was 20.0 months (95% CI: 17.8-22.1).^4^ Given this data, the US FDA has granted accelerated approval to pembrolizumab in combination with trastuzumab, fluoropyrimidine, and platinum chemotherapy in metastatic HER2-positive gastric or gastroesophageal junction adenocarcinoma, and this recommendation is now in the consensus guidelines from the National Comprehensive Cancer Network.

**Table 1.** Baseline characteristics.

Characteristic	All subjects (*n* = 18)
Median age (range)	63 (46-76)
Sex (%)	
Male	13 (72%)
Female	5 (28%)
Race and ethnicity (%)	
White, non-Hispanic	11 (61%)
White, Hispanic, or Latino	4 (22%)
Black/African-American	1 (6%)
Asian	2 (11%)
Primary tumor site	
Esophagus	4 (22%)
Gastroesophageal junction	8 (44%)
Stomach	4 (22%)
Not described or multiple	2 (11%)
HER2 IHC (%)	
3+	12 (67%)
2+ with ISH+	6 (33%)
PD-L1 CPS score (%)	
0	0 (0%)
1 to <5	3 (17%)
5 to <10	4 (22%)
≥10	2 (11%)
Unknown	9 (50%)

**Table 2.** Best response rate by 24 weeks.

	Best response rate by 24 weeks (confirmed or unconfirmed)	Best confirmed response rate
Response	Number (%) of subjects (*n* = 18)	Number (%) of subjects (*n* = 18)
Complete response	1 (6)	0 (0)
Partial response	10 (56)	9 (50)
Stable disease	5 (28)	7 (39)
Progression	2 (11)	2 (11)

**Table 3.** Grade 3-4 treatment-related adverse events.

Treatment-related grade 3-4 AEs	Number (%) of subjects (*n* = 18)
Hematologic	
Neutrophil count decreased	6 (33)
Platelet count decreased	2 (11)
Anemia	2 (11)
White blood cell decreased	1 (6)
Lymphocyte count decreased	1 (6)

Non-hematologic	
Hypokalemia	2 (11)
Diarrhea	2 (11)
Gastroesophageal reflux disease	1 (6)
Lung infection	1 (6)
Mucositis oral	1 (6)

Notably, while FOLFOX is very commonly used as a chemotherapy backbone in gastroesophageal cancers, this regimen was not included in KEYNOTE-811, and our study provides prospectively collected data from a clinical trial showing feasibility and promising activity with adding an immune checkpoint inhibitor specifically to the FOLFOX regimen along with trastuzumab. The toxicity profile in our study did show higher rates of grade 3-4 hematologic toxicities, such as neutropenia (33%) and thrombocytopenia (11%), compared to KEYNOTE-811 with grade 3-4 neutropenia (8%) and thrombocytopenia (6%). The hematologic toxicities tended to be higher, likely due to the 5-fluorouracil bolus in mFOLFOX6, and other studies of FOLFOX backbone in gastroesophageal cancers have shown comparable rates of grade 3-4 hematologic toxicities.^5,6^ In our study, no patients had grade 3-4 peripheral sensory neuropathy, but 61% of patients had grade 1-2 sensory neuropathy, while in KEYNOTE-811, there were 4% of patients with grade 3-4 peripheral sensory neuropathy and 24% of patients with any grade of peripheral sensory neuropathy.

Our study also evaluated this regimen across a range of subgroups, including patients whose tumors were HER2 positive by in situ hybridization (ISH+) but had lower HER2 protein expression of 2+. Of the 6 patients with HER2 2 + disease, 3 (50%) had progressive disease, 3 (50%) had stable disease as best response, a median PFS of 6.88 months (95% CI: 4.34-NA), and a median OS of 9.46 months (95% CI: 4.34-NA) compared with 12 patients with HER2 3 + disease, of whom 6 (50%) had partial response, 4 (33%) had stable disease, and 2 (17%) had progressive disease as best response. Though our study was limited by 50% of samples having unknown PD-L1 expression level, we did observe responses among patients whose tumors had relatively low PD-L1 expression with CPS as low as 2; no patients enrolled in our study had tumors known to have PD-L1 CPS < 1. Prior studies have shown comparable PFS across a range of PD-L1 expression^1,2^ and HER2 IHC levels.^1^ However, KEYNOTE-811 data does demonstrate that the subgroup of patients whose tumors had CPS ≥ 1 derived most of the improvement in PFS with addition of anti-PD1 therapy with hazard ratio (HR) 0.71 (95% CI: 0.59-0.86), while patients whose tumors had CPS < 1 did not have significant improvement in PFS, with HR 1.03 (95% CI: 0.65-1.64).^4^ This data resulted in a revision of the FDA accelerated approval for the addition of pembrolizumab to chemotherapy plus trastuzumab only in patients whose tumors also have PD-L1 CPS ≥ 1. HER2 non-amplified status was associated with worse PFS in prior studies,^1,3^ and so this may be a relevant biomarker for activity as well that merits further study. Samples collected from this study, including serial circulating tumor DNA (ctDNA) samples and stool for gut microbiome analyses, will serve as the source for additional impactful correlative studies to further identify biomarkers of response.

However, our study has several limitations. Most importantly, the small sample size and the early discontinuation of our clinical trial before the intended second stage of the design preclude the intended hypothesis testing as compared to historical control from ToGA, and also widen the confidence interval of estimates of PFS, OS, and ORR.

While the results from our study were encouraging, the confirmed ORR of 50% was lower than observed in the other studies of chemotherapy, trastuzumab, and pembrolizumab, including importantly the KEYNOTE-811 trial. There are several possibilities that could underlie this difference. The choice and timing of maintenance therapy may have some impact; our trial prespecified deescalating to trastuzumab + avelumab maintenance, without any cytotoxic therapy, after completing 9 cycles of induction chemotherapy, which is common in clinical practice to maximize patient quality of life, and was the strategy described in the ToGA^7^ and the HELOISE trials^8^; as these trials formed the basis for our alternative hypothesis, we chose to limit chemotherapy to 18 weeks as described in those studies. However, it is possible that continuing fluoropyrimidine maintenance could have extended the duration of disease control. In KEYNOTE-811, patients were allowed to continue the full chemotherapy backbone or the fluoropyrimidine alone, in addition to trastuzumab and pembrolizumab versus placebo, for up to 35 cycles, and this difference in chemotherapy backbone may have contributed to differences in outcomes. Additionally, our study used the PD-L1 antibody avelumab, as opposed to the PD-1 antibody pembrolizumab. A recent systematic review and meta-analysis showed that anti-PD-1 antibodies were associated with improved OS and PFS compared to anti-PD-L1 antibodies, including in gastric cancer (OS HR 0.57, 95% CI: 0.42-0.78).^9^ While the mechanism of this difference is not clear, it is possible that the failure of PD-L1 antibodies to block PD-L2 binding to PD-1 may allow for resistance to PD-L1 antibodies when there is high PD-L2 expression, and gastric cancers have been shown to have moderate to high expression of PD-L2.^10^ Avelumab has been studied in metastatic gastric cancer as a single-agent in the third-line setting (JAVELIN Gastric 300) and as maintenance after induction first-line chemotherapy (JAVELIN Gastric 100), and these studies did not meet their primary endpoints.^11,12^ These studies were not biomarker-selected, and the avelumab was not administered in combination with other therapies, which likely contributed to the negative results; however, it is possible that the mechanism and target of the immune checkpoint inhibitor do impact efficacy. Ultimately, the ORR, PFS, and OS from KEYNOTE-811 were superior to the results from our study, and possible contributing factors are the limited sample size, the lack of cytotoxic chemotherapy during maintenance, and the use of the anti-PD-L1 antibody rather than anti-PD1 in our study.

In conclusion, the addition of avelumab to trastuzumab and FOLFOX had clinically meaningful ORR and PFS in previously untreated metastatic HER2-overexpressing gastric and esophageal adenocarcinomas, corroborating other trials showing activity of immune checkpoint inhibitors added to trastuzumab and fluoropyrimidine/platinum chemotherapy regimens. While the results of KEYNOTE-811 have established pembrolizumab as the immune checkpoint inhibitor with the strongest level data and the current standard of care in combination with trastuzumab and doublet chemotherapy, our study does provide additional context stressing the continuation of maintenance chemotherapy and provides data on the FOLFOX chemotherapy backbone in combination with trastuzumab and PDL1 inhibition.

## Data Availability

The datasets, including the study protocol, statistical analysis plan, and individual participants’ data supporting the results reported in this article, will be made available from the completed study within 3 months from initial request to researchers who provide a methodologically sound proposal. The data will be provided after its de-identification, in compliance with applicable privacy laws, data protection, and requirements for consent and anonymization.
